# *citSATdb*: Genome-Wide Simple Sequence Repeat (SSR) Marker Database of *Citrus* Species for Germplasm Characterization and Crop Improvement

**DOI:** 10.3390/genes11121486

**Published:** 2020-12-10

**Authors:** Naveen Duhan, Manish Meshram, Cristian D. Loaiza, Rakesh Kaundal

**Affiliations:** 1 Department of Plants, Soils and Climate, Utah State University, Logan, UT 84322, USA; naveen.duhan@aggiemail.usu.edu (N.D.); cdloaiza@aggiemail.usu.edu (C.D.L.); 2 Center for Integrated BioSystems (CIB), Utah State University, Logan, UT 84322, USA; 3 Department of Computer Science, Utah State University, Logan, UT 84322, USA; manish.meshram@aggiemail.usu.edu

**Keywords:** SSR, citrus, marker-assisted selection, database, microsatellites, genomics, plant breeding and genetics

## Abstract

Microsatellites or simple sequence repeats (SSRs) are popular co-dominant markers that play an important role in crop improvement. To enhance genomic resources in general horticulture, we identified SSRs in the genomes of eight citrus species and characterized their frequency and distribution in different genomic regions. *Citrus* is the world’s most widely cultivated fruit crop. We have implemented a microsatellite database, *citSATdb*, having the highest number (~1,296,500) of putative SSR markers from the genus *Citrus*, represented by eight species. The database is based on a three-tier approach using MySQL, PHP, and Apache. The markers can be searched using multiple search parameters including chromosome/scaffold number(s), motif types, repeat nucleotides (1–6), SSR length, patterns of repeat motifs and chromosome/scaffold location. The cross-species transferability of selected markers can be checked using e-PCR. Further, the markers can be visualized using the Jbrowse feature. These markers can be used for distinctness, uniformity, and stability (DUS) tests of variety identification, marker-assisted selection (MAS), gene discovery, QTL mapping, and germplasm characterization. *citSATdb* represents a comprehensive source of markers for developing/implementing new approaches for molecular breeding, required to enhance *Citrus* productivity. The potential polymorphic SSR markers identified by cross-species transferability could be used for genetic diversity and population distinction in other species.

## 1. Introduction

The genetic selection of plants in conventional plant breeding is decided by the parents and influenced by different environmental conditions [1]. In conventional plant breeding, alleles are mixed over the generations, resulting in the development of new combinations, which helps in achieving higher trait value through selection. Developing a variety of woody plant species through traditional breeding may take 1012 years [2]. This time period can be reduced by performing marker-assisted selection (MAS) on seedling material [3]. In recent years, marker-assisted selection has become popular in breeding programs for many crops [4,5,6,7]. One of the pre-requisites for using MAS is the discovery of DNA-based markers, which are tightly linked to the target trait of interest. Microsatellite (SSR) markers have been the system of choice for quantitative trait loci (QTL) mapping in many crops for a long time. Microsatellites are tandem repeats of 1–6 nucleotide long DNA units flanked by unique sequences in the genome but found more abundantly in the intronic region [8]. These are characterized by multi-allelic variation, reproducibility, and a high co-dominant inheritance [9]. The mutation rate of SSRs ranges from 10^−3^ to 10^−6^ per generation [10], which increases with the length of the repeat unit [11]. They are highly versatile, low-cost, informative PCR-based molecular markers associated with a high frequency of length polymorphism [12]. These features make them the preferred choice among available genetic markers (e.g., AFLP, RAPD, RFLP, SNP and SRAP) and provide the basis for their effective applications in a wide range of fields, such as genetic mapping, QTLs identification, varietal identification, genetic diversity analysis, linkage mapping, marker-assisted selection and evolutionary analysis [13].

The genus *Citrus* is a large taxonomic rank that includes many cultivated species such as oranges, lemons, pomelos, grapefruits, and limes. *Citrus* plants are woody, perennial small to moderate-sized trees that are cultivated all over the world to produce fresh fruits and juice, and as ornamentals, etc. Being part of our heritage, the citrus industry has a great social and cultural significance, in addition to its economic and agronomic importance. A number of SSR markers have been reported in citrus in different studies, but there is a limited catalogue. Therefore, there is a need for more comprehensive identification of markers for genetic diversity, MAS, association or comparative mapping, genetic linkage maps, and qualitative and quantitative traits [14,15,16,17,18,19,20,21,22].

Conventional methods for SSR screening using genomic libraries are costly, labor-intensive and time-consuming [23]. In silico approaches can thus be used to overcome this problem. These methods have the advantage of predicting SSRs in specific regions in the genome, which is more efficient in designing molecular markers for linkage mapping and QTLs [24]. The advent of next-generation sequencing (NGS) technologies and advancement in computational approaches have made possible the discovery of markers in bulk.

In accordance with the TRIPS (trade-related aspects of intellectual property rights) agreement and other intellectual property rights, plant breeders own a variety on the basis of distinctness, uniformity and stability (DUS) characteristics, which can be used for allocating new variety status and solving legal disputes [25]. So, in order to supplement DUS characteristics for variety identification, SSR markers were successfully used in crops such as rice [26], maize [27], barley [28], tobacco [29], soybean [30], wheat [31], mung beans [32], kadam [33] and potatoes [34]. The characterization of different *Citrus* species can also be undertaken using such approaches. The *Citrus* genus contains more than 100 species with limited genomic resources available. Extensive molecular mining of SSR markers and assessment of their polymorphism with cross-species transferability may be a more pragmatic approach to addressing the need for markers in previously untouched species. In closely related species, flanking regions of SSRs are conserved; heterologous primers of these flanking regions can facilitate the use of molecular markers [35]. SSR markers from focal species can be applied in non-focal species to investigate the population genetic structure of wild species [36].

Though different microsatellite databases have been developed [37,38], due to the high number of species in these databases, they lack some features, such as e-PCR, browsing/visualizing SSRs on the genome, etc. Therefore, in addition to the existing databases, a dedicated database focusing on all available genomic data of the genus *Citrus* that contains a wide range of user-friendly features could be a valuable genomic resource for *Citrus* crop improvement and characterization.

The present study was aimed at genome-wide mining of SSRs and the development of a user-friendly database containing microsatellites from eight *Citrus* species (*Citrus sinensis*, *Citrus clementina*, *Citrus maxima*, *Citrus medica*, *Citrus ichangensis*, *Atlantia buxifolia, Citrus reticulata* and *Fortunella hindsii*) with the options for chromosome-wise SSR mining and primer designing for genotyping, along with e-PCR-based polymorphism discovery. It also aims to provide annotated genic regions of SSR to be used as functional domain markers (FDMs).

## 2. Materials and Methods

### 2.1. Data Collection

The genomes of *Citrus sinensis* [39], *Citrus clementina* [40], *Citrus maxima*, *Citrus medica*, *Citrus ichangensis*, and *Atlantia buxifolia* [41] were downloaded from the *Citrus* Genome Database [42]; *Citrus sinensis* and *Citrus maxima* were assembled chromosome-wise while *Citrus medica*, *Citrus clementina*, *Citrus ichangensis*, and *Atalantia buxifolia* were assembled scaffold-wise, *Citrus reticulata* through pseudomolecule assembly and *Fortunella hindsii* through contig-assembly (Table 1). Genome assemblies were used from these two resources: Huazhong Agricultural University (HZAU), and Joint Genome Institute (JGI).

### 2.2. In Silico Simple Sequence Repeat Mining and Primer Designing

SSRs were identified in genomes of 8 citrus species (Table 1). A Perl script (*miSATminer*) was written to identify repeat motifs in a genome sequence. Microsatellites were identified with parameters such as 10 repeat units for mono, 5 repeat units for the di, tri, tetra, penta, and hexa. In-house Perl scripts were used to fetch the flanking regions of the identified SSRs for primer designing. Primer3 executables were used to design primers with the following default parameters: melting temperature, 55–65 °C; GC content, 40–60%; primer size, 18–27 bp; length and product size, 150–280 bp [43].

### 2.3. Functional Annotation of SSR Markers

The full annotation of gene functions is available for these eight citrus species and was implemented in the *Jbrowse* genome browser inside the *citSATdb* database. In this genome browser, markers can be visualized against the reference sequence, gene coordinates, and structural and functional details.

### 2.4. Marker and Database Development Workflow

**Figure 1:**
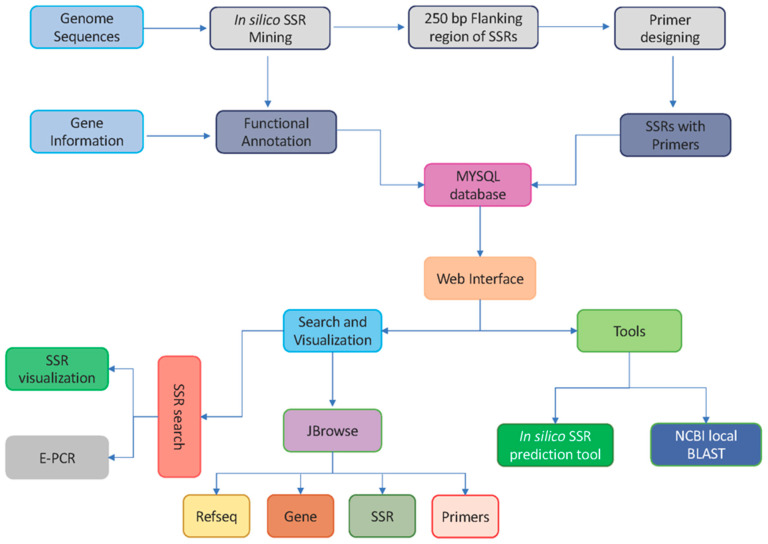
The schematic workflow of *citSATdb* (citrus microSATellite) database.

Microsatellite repeat loci were mined by pattern identification in the genome sequences using *miSATminer*, our inhouse developed Perl script. This script mines SSR loci from the genome sequences with custom repeat parameters. SSR primers for genotyping were designed using Primer3 executables by extracting a flanking length of 300 bp upstream and 300 bp downstream of SSR loci in the genome. Selected repeats can be viewed with their markers in the sequence. ePCR was implemented in the database for polymorphism. In *citSATdb*, we have provided eight genome assemblies and an option for uploading user sequences to check amplification. The markers with variable product size in two genomes were considered as polymorphic markers. All the results can be downloaded as a CSV file. The whole workflow of the database is depicted in (Figure 1).

### 2.5. Database Development and Web Interface

The *Citrus* microsatellite database (*citSATdb*) is a three-tier-based relational database developed with a client tier, middle tier, and database tier. Predicted SSRs and their corresponding primers were stored in MySQL data tables and accessed through the Apache server. A user-friendly interface of the database was developed with PHP, HTML5, and Jquery. In silico microsatellite designing with *miSATminer* and custom Perl scripts and Primer3 was implemented for primer designing. Jbrowse for the visualization of genomic sequences, SSRs and primers was also implemented. The NCBI local and remote database was also implemented for similarity searches. e-PCR was implemented for cross-species transferability. The web server contains seven tabs *viz*. Home, About, Species, Tools, JBrowse, Help, and Contact; the database will be updated regularly with newly available genome data.

## 3. Results and Discussion

### 3.1. Cross-Species Comparison of Citrus Species SSRs

For the development of the *Citrus* web genomics resource, SSR loci were mined successfully using miSATminer. A total of 1,699,853 putative microsatellites were mined from the genomes of eight *Citrus* species. The highest number of microsatellites were identified in *Fortunella hindsii* (240,182), followed by *Citrus ichangensis* (226,950), *Citrus maxima* (224,961), *Citrus medica* (210,590), *Atalantia buxifolia* (204,687), *Citrus sinensis* (203,297), *Citrus reticulata* (201,408) and *Citrus clementina* (187,778). Maximum microsatellite density (SSRs/Mb) was observed in *Atalantia buxifolia* (675.26), whereas the minimum was observed in *Citrus medica* (552.12) (Table 2). Previous studies have reported a negative correlation between the SSR density and genome size [44]. However, the SSRs identified in our study of eight *Citrus* species show no correlation between the SSR density and genome size. This is in line with some of the recent findings which reported that there is no correlation between the genome size and SSRs density; genome size differences may lead to the degree of microsatellite repetition in the genome [45,46,47,48,49,50].

### 3.2. SSR Motifs Characterized by Repeat Length

**Figure 2:**
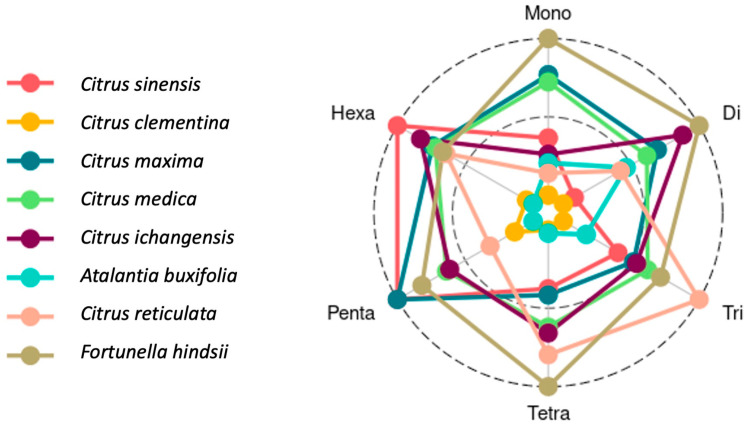
Distribution of SSR motifs in each of the *Citrus* species.

In all the species, mono-nucleotide repeats were most abundant, followed by di-, tri-, tetra-, penta-, and hexa-nucleotide repeats. Among all the citrus species, the maximum number of mono-nucleotide repeats was found in *Fortunella hindsii* (152,611) followed by *C. ichangensis* (144,115), *C. maxima* (142,446), *A. buxifolia* (129,304), *C. medica* (125,467), *C. reticulata* (123,469), *C. sinensis* (121,051), and *C. clementina* (115,888). In the case of di-nucleotide repeats, the maximum number was observed in *F. hindsii* (61,408) followed by *C. medica* (60,040), *C. ichangensis* (57,930), *C. maxima* (57,059), *C. reticulata* (55,336), *C. sinensis* (54,874), *A. buxifolia* (51,030), and *C. clementina* (50,108). The occurrence of tri-nucleotides was observed highest in *C. sinensis* (23,568) followed by *F. hindsii* (22,143), *C. maxima* (21,668), *C. medica* (21,259), *C. ichangensis* (21,149), *A. buxifolia* (20,572), *C. reticulata* (19,400), and *C. clementina* (18,553). Similarly, tetra-nucleotides were most frequent in *F. hindsii* (3159) followed by *C. sinensis* (3050), *C. medica* (2975), *C. maxima* (2954), *C. ichangensis* (2844), *A. buxifolia* (2822), *C. reticulata* (2631), and *C. clementina* (2620). *C. ichangensis* (613) and *A. buxifolia* (613) contain the maximum penta-nucleotides, followed by *F. hindsii* (576), *C. maxima* (539), *C. medica* (534), *C. sinensis* (473), *C. clementina* (436), and *C. reticulata* (408). Hexa-nucleotides were most abundant in *A. buxifolia* (346) followed by *C. medica* (315), *C. ichangensis* (299), *C. maxima* (295), *F. hindsii* (285), *C. sinensis* (281), *C. clementina* (173), and *C. reticulata* (164) (Table 3, Figure 2). From these results, a high abundance of mono-nucleotide repeats was observed in all the genomes, which may be due to the intrinsic limitation of the chemistry of next-generation sequencing (NGS) technology used for data generation [51]. Similarly, di-nucleotide repeats in higher abundance have also been reported in other crops [52,53].

### 3.3. Designed SSR Primers, Motif Characterization by Repeat Length

**Figure 3:**
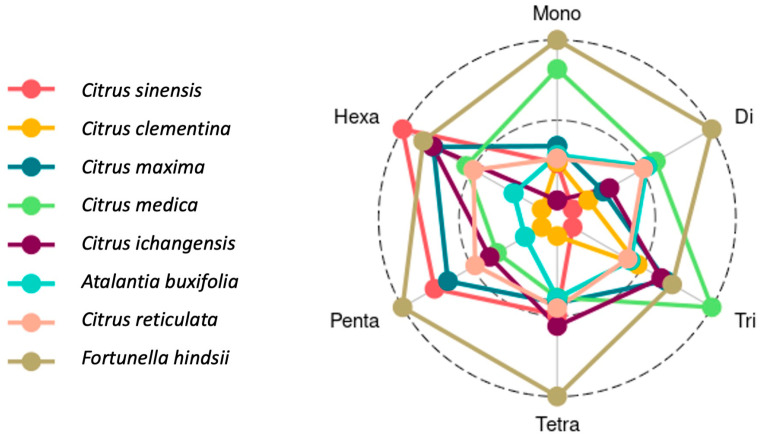
Distribution of motif types in the predicted SSR markers.

*citSATdb* is a comprehensive microsatellite database of *Citrus* represented by eight species containing 1,296,500 in silico predicted markers. Distribution-wise, mononucleotide repeat primers were the most abundant followed by di-, tri-, tetra-, penta-, and hexa-nucleotide. Among the eight species, the maximum number of mononucleotide repeat primers were designed in *F. hindsii* (128,597) followed by *C. maxima* (120,885), *C. ichangensis* (100,422), *C. reticulata* (98,016), *C. sinensis* (97,149), *C. clementina* (96,191), *A. buxifolia* (95,905), and *C. medica* (85,972). In the case of di-nucleotide primers, the maximum number was observed in *F. hindsii* (50,791) followed by *C. maxima* (45,399), *C. reticulata* (44,528), *C. sinensis* (44,198), *C. medica* (40,922), *C. ichangensis* (40,333), *C. clementina* (38,870), and *A. buxifolia* (37,363). The occurrence of tri-nucleotides motif primer was observed highest in *C. maxima* (16,037) followed by *F. hindsii* (14,639), *C. ichangensis* (14,404), *C. medica* (14,248), *C. clementina* (13,418), *C. reticulata* (13,171), *C. sinensis* (13,069), and *A. buxifolia* (11,120). Similarly, tetra-nucleotides primers were most frequent in *F. hindsii* (2390) followed by *C. medica* (2097), *A. buxifolia* (2050), *C. sinensis* (2022), *C. ichangensis* (2002), *C. reticulata* (1981), *C. maxima* (1979), and *C. clementina* (1722). *F. hindsii* (494) contains maximum penta-nucleotides primers followed by *A. buxifolia* (446), *C. ichangensis* (426), *C. sinensis* (385), *C. medica* (363), *C. maxima* (352), *C. reticulata* (310)*,* and *C. clementina* (285). Hexa-nucleotides primers were most abundant in *A. buxifolia* (256) followed by *F. hindsii* (234), *C. medica* (224), *C. ichangensis* (223), *C. maxima* (188), *C. sinensis* (180), *C. reticulata* (137), and *C. clementina* (107) (Table 4, Figure 3).

The designed SSR primers can be used for QTL/candidate gene identification, linkage mapping, and germplasm characterization. Varieties with similar morphological characteristics are very difficult to differentiate from just the phenotypic study. To conquer these difficulties, SSR markers have been used in previous studies for variety characterization, trait improvement, linkage mapping, molecular breeding application, variety development, and phylogenetic and taxonomic comparisons [8,53,54,55]. Similarly, 24 SSR markers were used to assess genetic diversity in 370 *Citrus* accessions [19]. The designed putative primers present in *citSATdb* can be used in rapid genotyping for genetic diversity and differentiating varieties. Varietal differentiation using SSR markers has already been reported in many other crops, such as barley [56], sugarcane [57], eggplant [58], capsicum [59], and sesame [60]. These markers can be further explored for trait improvement averse to abiotic and biotic stresses. For example, in Satsuma mandarins, SSR has been used to discover one major QTL for male sterility, and such a QTL can be used in seedless citrus breeding by using flanking region SSR markers with allele size differences between donor and recipient varieties [61]. Such markers can be used for high-density linkage mapping and the discovery of genes needed to improve specific traits. Using SSRs, a linkage map was developed, and QTL mapping was performed to find loci related to the freezing tolerance of citrus [62].

The availability of whole-genome assemblies of different plant species in the public domain provides an opportunity for the study of cross-species transferability in closely related species. Trait-specific candidate genes may be cloned from different species [63]. In silico cross-species transferability can also be predicted with *citSATdb*, which can be further used for phylogenetic and diversity studies. A similar use has been reported for diversity analysis in citrus species with few numbers of markers [19].

### 3.4. Functional Annotation of SSRs and Markers

All the predicted SSRs were mapped on the gene feature file (GFF) of each genome. In *C. sinensis*, 62,563 SSRs were found to be mapped onto the genic regions, followed by *C. clementina* (45,975), *C. maxima* (60,654), *C. medica* (49,403), *C. ichangensis* (52,336), *A. buxifolia* (53,937), *C. reticulata* (53,832) and *F. hindsii* (73,901) (Table 5). Further, we designed primers for each of the species; in *C. sinensis* (48,804), *C. clementina* (42,476), *C. maxima* (60,267), *C. medica* (50,690), *C. ichangensis* (54,065), *A. buxifolia* (46,373), *C. reticulata* (48,203), and *F. hindsii* (67,288), genic SSR primers were designed. A detailed distribution of the predicted and designed markers on both the genic and non-genic regions is presented in (Table 5).

### 3.5. Comparison with Another Databases

Many databases of marker development in plants are publicly available. The Pan-Species Microsatellite Database (*PSMD)* database contains eight *Citrus* species in its repository, although it lacks some features such as e-PCR, JBrowse, and BLAST. Plant micro-satellite Database (*PMDbase*) is another online database, but it has some limitations such as the markers search by user choice, repeat kind, motif type, location in the genome, etc. Secondly, only two species of citrus are present in this database. Similarly, SSRome also has only two *Citrus* species and lacks features such as ePCR, JBrowse, BLAST, etc. The *citSATdb* resource overcomes these limitations and is specifically designed as a user-friendly interface to assist the researchers in the horticultural sciences. A detailed comparison of *PMDBase*, PSMD, SSRome, and *citSATdb* is presented in (Table 6).

### 3.6. citSATdb: Citrus Microsatellite Web-Genomic Resource

**Figure 4:**
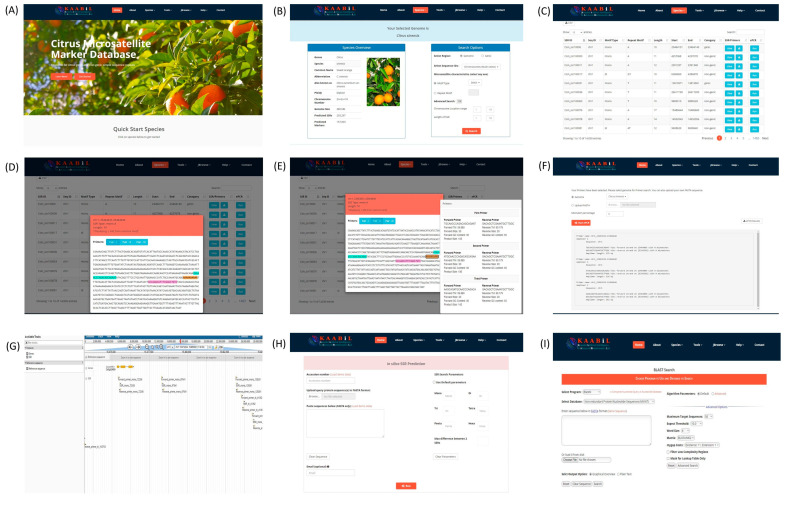
The main functions of the *citSATdb* database and search modules. (**A**) Home page; (**B**) species search page; (**C**) results page showing the desired search results; (**D**) results page displaying SSR and corresponding primers in sequence; (**E**) sequence of primers and their features; (**F**) ePCR results page; (**G**) JBrowse for visualization of markers on genome; (**H**) *miSATminer* tool for SSR prediction; and (**I**) BLAST search page.

The citrus web-genomic resource (*citSATdb*) was developed successfully using a three-tier architecture. This is a comprehensive microsatellite database of *Citrus* represented by eight species containing 1,296,500 in silico predicted markers. The web server contains seven tabs *viz*. Home, About, Species, Tools, JBrowse, Help, and Contact. The ‘Species’ tab provides information about the selected species on left and search options on the right side. In silico predicted markers can be searched by selecting genic or genomic, chromosome/scaffold-wise, along with motif type, repeat type, length, and location in the genome. The search results provide a visualization of repeat and flanking primers on the sequence extracted with 500 bp upstream and downstream of the repeat. It also provides an option for ePCR whereby users can check the in silico amplification of selected primers in the genome or cross-species transferability with the user-given sequence(s). All the results can be downloaded in a CSV format text file. The ‘Tool’ page provides two tabs—SSR prediction and BLAST. *miSATminer* was implemented with custom scripts to design SSRs and their primers for user input sequences. Standalone BLAST was implemented on the BLAST search page, where users can align their SSR query sequences to genomes. All the eight genome sequences can be visualized with gene and SSR coordinates on the genome using the ‘JBrowse’ table. The ‘Help’ tab contains a detailed tutorial for using the database efficiently and a list of frequently asked questions. A detailed workflow of exploring the *citSATdb* and its search features is illustrated in Figure 4.

## 4. Conclusions

We report here a comprehensive web genomic resource for the genus *Citrus* covering three of its commercially important species. *citSATdb*, accessed freely via the address http://bioinfo.usu.edu/citSATdb/, contains a total of 1,296,500 putative microsatellite DNA markers. Our findings on the cross-species transferability of microsatellite loci among six different species of *Citrus* can be used to cater to the need for molecular markers, especially for the more than 100 species of the genus *Citrus* for which there are no whole-genome sequence data available yet. This genomic resource can be of immense use to the global community. It can be used for chromosome-wise microsatellite locus mining and primer designing for non-genic and genic FDM-SSR for rapid genotyping. It can also be used to accelerate polymorphism discovery by e-PCR, thus being economically beneficial and needed in future re-sequencing projects. The database can be used not only for knowledge discovery research, such as QTL and gene mapping, but also for marker-assisted breeding in *Citrus* germplasm improvement and management.

## Figures and Tables

**Table 1:** The genomic data of different *Citrus* species used in the study.

Species	Assembly Version	Assembly Level	Genome Size (Mb)	GC%
*Citrus sinensis*	v2.0 (HZAU)	Chromosome	327.945	34.06%
*Citrus clementina*	v1.0 (JGI)	Scaffold	301.387	34.96%
*Citrus maxima*	v1.0 (HZAU)	Chromosome	345.78	34.99%
*Citrus medica*	v1.1 (HZAU)	Scaffold	406.058	35.16%
*Citrus ichangensis*	v1.0 (HZAU)	Scaffold	357.621	34.21%
*Atalantia buxifolia*	v1.1 (HZAU)	Scaffold	315.821	33.55%
*Citrus reticulata*	v1.1(HZAU)	Pseudomolecule	334.2	-
*Fortunella hindsii*	V1.1(HZAU)	Contig	373.6	34.49%

**Table 2:** Distribution of identified SSRs in *Citrus* species.

Species	Predicted SSRs	Designed Markers	SSRs/MB	Genome Size (MB)
*Citrus sinensis*	203,297	157,003	619.91	380
*Citrus clementina*	187,778	150,593	638.15	370
*Citrus maxima*	224,961	184,840	650.59	328
*Citrus medica*	210,590	143,826	552.12	380
*Citrus ichangensis*	226,950	157,810	648.06	407
*Atalantia buxifolia*	204,687	147,140	675.26	391
*Citrus reticulata*	201,408	158,143	602.66	370
*Fortunella hindsii*	240,182	197,145	642.89	370

**Table 3:** Distribution of predicted SSR motif types in each of the *Citrus* species.

Species	Mono	Di	Tri	Tetra	Penta	Hexa
*Citrus sinensis*	121,051	54,874	23,568	3050	473	281
*Citrus clementina*	115,888	50,108	18,553	2620	436	173
*Citrus maxima*	142,446	57,059	21,668	2954	539	295
*Citrus medica*	125,467	60,040	21,259	2975	534	315
*Citrus ichangensis*	144,115	57,930	21,149	2844	613	299
*Atalantia buxifolia*	129,304	51,030	20,572	2822	613	346
*Citrus reticulata*	123,469	55,336	19,400	2631	408	164
*Fortunella hindsii*	152,611	61,408	22,143	3159	576	285

**Table 4:** Distribution of motif types in the designed SSR markers for each of the *Citrus* species.

Species	Mono	Di	Tri	Tetra	Penta	Hexa
*Citrus sinensis*	97,149	44,198	13,069	2022	385	180
*Citrus clementina*	96,191	38,870	13,418	1722	285	107
*Citrus maxima*	120,885	45,399	16,037	1979	352	188
*Citrus medica*	85,972	40,922	14,248	2097	363	224
*Citrus ichangensis*	100,422	40,333	14,404	2002	426	223
*Atalantia buxifolia*	95,905	37,363	11,120	2050	446	256
*Citrus reticulata*	98,016	44,528	13,171	1981	310	137
*Fortunella hindsii*	128,597	50,791	14,639	2390	494	234

**Table 5:** Distribution of SSRs in the individual *Citrus* genome.

Species	Predicted Markers		Designed Markers	
	Genic	Non-Genic	Genic	Non-Genic
*Citrus sinensis*	62,563	140,734	48,804	108,199
*Citrus clementina*	45,975	141,803	42,476	108,117
*Citrus maxima*	60,654	164,307	60,267	124,573
*Citrus medica*	49,403	161,187	50,690	93,136
*Citrus ichangensis*	52,336	174,314	54,065	103,745
*Atalantia buxifolia*	53,937	150,750	46,373	100,767
*Citrus reticulata*	53,832	147,576	48,203	109,940
*Fortunella hindsii*	73,901	166,281	67,288	129,857

**Table 6:** Comparison of *citSATdb* with PMDBase, PSMD, and SSRome features.

Features	citSATdb	PMDBase	PSMD	SSRome
*Citrus* species	8	2	8	2
Search option with ID	√	×	√	×
Search by motif type	√	×	√	√
Search by repeat type	√	×	√	√
Genic and genomic search	√	×	√	√
Search on user-defined location	√	×	√	×
Length of SSR	√	×	√	×
ePCR option	√	×	×	×
Non-nuclear (mitochondrion and chloroplast)	×	√	×	×
JBrowse visualization of SSRs	√	×	×	×
BLAST	√	×	×	×
